# Impact of Lockdown due to COVID-19 on lifestyle and diet pattern of college students of Eastern India: A cross-sectional survey

**DOI:** 10.3126/nje.v12i1.42292

**Published:** 2022-03-31

**Authors:** Santosh Kumar Nirala, Bijaya Nanda Naik, Rajath Rao, Sanjay Pandey, CM Singh, Neha Chaudhary

**Affiliations:** 1-6 Department of Community and Family medicine, All India Institute of Medical Sciences, Patna, India

**Keywords:** COVID-19, Dietary habits, Lifestyle, Non-communicable diseases, Physical activity

## Abstract

**Background:**

The emergence of the COVID-19 pandemic and lockdown measures to confine it has disrupted the routine of the public. The impact of such long-term confinements on the lifestyle and diet of students are not known and hence this study was designed to assess the impact of lockdown due to the COVID-19 pandemic on the lifestyle and diet of university students.

**Methods:**

An online cross-sectional survey among 622 university students across various educational institutes of east India using a pre-designed questionnaire about lifestyle-diet before and during the lockdown. Results were tabulated and statistical tests like Paired t-test, Wilcoxon Rank sign test, and Mc-Nemar tests were applied and overall significance was attributed to P<0.05.

**Results:**

During the lockdown a total of 2.4% (95% CI: 1.4-3.8%) decrease in prevalence of tobacco use, 8.7% (95% CI: 6.6-11%) decrease in physical activity and a 0.8 hour (95% CI: 0.6-0.9 hour) increase in the mean sleep duration was observed. There was a significant increase in use of fresh fruits consumption [Median(IQR)-before:2(1-5);during:3(1-5) days] and a decrease in meat-poultry[Median(IQR)-before: 2(0-3);during: 1(0-3)days] and junk food[Median(IQR)-before:1(0-2);during:0(0-2)days] consumption during the lockdown.

**Conclusion:**

A significant proportion of changes in lifestyle and frequency of consumption of certain food items in the dietary pattern during the lockdown.

## Introduction

COVID-19 was declared a pandemic on March 11, 2020 [1]. Since then, countries have implemented rigorous measures to restrict the disease spread, including social distancing, mandatory mask use, travel bans, and strict and partial lockdowns with non-essential services prohibited. The Government of India announced the first nationwide lockdown on March 24, 2020, for 21 days, limiting the movement of the entire 1.3 billion population as a preventive measure against the pandemic [2,3]. The May 2021 lockdown had different phases in different states of India [4]. Many educational institutions including professional institutions were shut down. Measures such as “social distancing” and “work from home” are new normal in the pandemic era [5]. Once life begins to return to the normal post-COVID-19 pandemic, the impact of lockdown on the lifestyle, behavior patterns, psychological morbidity, stress, post-traumatic stress and its effect on lifestyle and eating pattern are still unclear [6].

Physical inactivity and sedentary behaviour are two more pandemics that the globe has been struggling with for a long time [7]. According to the WHO (World Health Organization), 31% of people aged 15 and over are physically inactive, and this unhealthy lifestyle is responsible for nearly 3.2 million deaths per year [8]. In an attempt to divert attention away from unpleasant emotions (e.g., stress, boredom) caused during a lockdown, people may develop problematic eating patterns. Such lifestyle changes, such as increased consumption of high-calorie items and decreased consumption of healthful foods like fresh fruits and vegetables, may have a significant impact on dietary patterns and quality [9].

Young people are more susceptible to lifestyle and dietary modifications than any other group affected by the COVID-19 lockdown [10]. Poor nutritional alterations made during a brief period of lockdown may lead to the development of unhealthy food preferences or behaviours in the medium to long term, affecting not only the teens’ health but also that of their spouses and children [11]. The educational institute’s closing has an impact on nutrition and lifestyle in numerous ways. Boredom is associated to consuming more energy than intended, while remaining at home (which includes online classes, limiting outdoor and gym physical exercise) and the disruption of work routine induced by the quarantine/confinement may cause boredom, which is linked to consuming more energy than intended [12]. Furthermore, limited access to daily grocery shopping may lead to a shift away from fresh foods, particularly fruits and vegetables, in favour of highly processed meals, such as junk foods, snacks, and ready-to-eat cereals, which are typically rich in fats, sugars, and salt.

Our health may be jeopardised by changes in our eating habits and lifestyle. Maintaining proper diet is critical, especially during a time when the immune system may be under attack. However, throughout the pandemic, there was a general decrease in physical activity, as well as an increase in the consumption of less healthful foods and sedentary behaviour [13,14]. Individuals with eating disorders and obesity may be affected by anxiety and uncertainty caused by the ongoing epidemic, as well as a loss in mental health owing to COVID-19, food insecurity, and limited healthcare access [15,16]. According to a review from Saudi Arabia, the pandemic’s side effects have created an adverse environment for healthy eating habits [17]. A review published in India emphasized lifestyle behavioral programs using e-Platform to improve the lifestyle of youth during such lockdowns [18].

There are limited studies done in India[2,19–22] showing the pattern of diet and lifestyle change among college students as an impact of lockdown due to ongoing pandemic. Therefore, the present study aimed to check the same among college students of east India with the objectives to find out the proportion of college students having a change in lifestyle and dietary pattern before and during lockdown to contain the spread of COVID-19.

## Methodology

### Study design and participants

#### Study design and participants

This cross-sectional study was conducted from May to July 2021 in online survey mode [23] across various educational institutions of east India which included medical, science, engineering, arts and commerce colleges namely AIIMS Patna, IGIMS Patna, NMCH Patna, Bhagalpur medical college, Gaya medical college and Narayan medical college-Rohtas. Science, Arts and commerce colleges were Patna women college, BS College Patna, College of Commerce Patna, Magadh University Gaya, Bhagalpur university. Law colleges were Chandragupta law university-Patna, Ranchi mahila college. Engineering college were NIT Jamshedpur, Chaibasa engineering college-Jamshedpur, MIT Muzaffarpur, Gaya engineering college and BC Roy engineering college-Durgapur

### Data collection

The information was collected using a pre-designed, semi-structured questionnaire in the form of Google form [24] and was sent to participants via WhatsApp and emails to complete. Digital consent was encrypted in the google forms link in the very beginning. Only after providing the consent, one can proceed further otherwise the participation ends.

### Questionnaire design and Validation

The questionnaire had four sections. Section A included basic demographic information about the respondents, section B included lifestyle changes and section C included questions about dietary changes before and during the lockdown, and section D included perception of students towards various changes about lifestyle and diet during the lockdown.

Lifestyle questions included tobacco use, physical activity, screen time, sleep pattern, sleep duration before and during lockdown while dietary questions included frequency of particular food/beverage per week before and during the lockdown. The last part included the perception of students towards various diet and lifestyle factors during lockdown which included change in body weight, sense of hunger, gadget use, fear, and anxiety towards COVID-19. The questionnaire emphasized the change in diet and lifestyle pattern one month before the lockdown and present-day during the lockdown when the questionnaire was administered.

The items of questionnaire initially when designed, was tested among the residents of the department and necessary changes were made and face validity was checked by three experts who were pioneers in the field of diet surveys, epidemiology and public health. Once finalized, the items were pretested among 40 students and internal consistency(reliability) in terms of Cronbach’s alpha of the items was found to be 0.90.

### Inclusion criteria

The study included all college-going students who are age >15 years

### Exclusion criteria

The students who refused voluntary participation in the survey were excluded.

### Sample size calculation

Considering the prevalence of change in sense of hunger and appetite during lockdown to be 52.2% [25] the minimum sample size was calculated to be 384 at 5% absolute precision and 95% confidence intervals (CI). The final sample size was calculated to be 427 after considering a non-response rate of 10% using Statulator [26].

The heads of certain educational institutions were contacted about the study and were requested to share the questionnaire among the students and later by snowball sampling[27], the students were requested to share the same among their peers to get the responses.

### Outcome variable

The primary outcome variable was to assess the pattern and change in lifestyle and dietary patterns of college students before and during the lockdown.

### Explanatory variables

Variables like age, gender, stream, batch, family status(above or below poverty line), body mass index(calculated using self-reported height and weight using the formulae for BMI calculation) were used to explain the sociodemographic information of the participants.

### Ethical committee approval

This study has been approved by Institute Ethics Committee, AIIMS, Patna (AIIMS/Pat/IEC/2020/737). We adhered to the principles of ethics throughout the study.

### Data management and Statistical analysis

Data was entered in MS Excel and analyzed using IBM SPSS Version 22 (SPSS Inc., Chicago, IL, USA) and the results were either tabulated or shown as figures if necessary. Descriptive analysis was conducted to describe the demographic characteristics. The categorical variables like tobacco use, physical activity, leisure time sports were expressed as frequency and percentages and continuous variables like frequency of consumption of food and beverage per week were expressed as median (IQR). For the ease of analysis, the frequency of food and beverage consumption per week was clubbed to ‘Never consumed’, ‘Some days a week’ (1-4days/week), and ‘often’ (5-7days/week) to find out the proportion of particular food and beverage consumption by students. The change in the proportion of lifestyle changes (Tobacco use, physical activity, leisure time sports) before lockdown and during lockdown was assessed by Mc Nemar test. The change in Sleep duration before and during lockdown was assessed by Paired T-test. The change in the diet and beverage changes before and during lockdown was assessed by Wilcoxon signed-rank test. The median (IQR) food and beverage consumption per week was represented as Box and whisker plot. The change in the proportion of major food groups and beverage consumption in a week before and during lockdown was assessed by the McNemar-Bowker test. Overall statistical significance was attributed to P-value <0.05.

## Results

Among 622 respondents, 397(63.8%) were male, 190(30.5%) were from science and 95(15.3%) from art backgrounds, 128(20.6%) belonged to the BPL(Below poverty line) category and 163(26.2%) were overweight and obese by calculating body mass index using self-reported height and weight. (Table 1)

### Habits

There was a statistically significant decrease in tobacco consumption during the lockdown, [9.2% before lockdown vs 6.8% during lockdown, P=0.01]. Also, physical activity reduced significantly during the lockdown. [58.8% before lockdown vs 50.2% during lockdown, P<0.001]

Also, there was a statistically significant increase of 0.8 hours of sleep before and during the lockdown.[Mean(SD)before lockdown:7.1(1.1) vs during lockdown:7.9(1.7) hours, p <0.001]. (Table 2)

### Sleep Patterns

Out of 622 students, 211(33.9%) had no change in the sleep pattern in lockdown compared to before lockdown. Almost half, 310(49.8%) reported that they slept late in the night while 96(15.4%) had excessive daytime sleeping. A total of 144(21.8%) reported having interrupted sleep and 42(6.8%) had difficulty falling asleep due to fear of COVID-19. (Table 3). Overall sleep quality was reported to be very good by 261(42.1%) students and very bad by 30(4.8%).

### Gadgets use

The median (IQR) screen time of students using gadgets during lockdown was 6(4-8) hours. Almost half, 331(53.2%) students used gadgets for ≥6hours (Screen time). Also, 240(38.6%) strongly agreed that their gadget dependence has increased during the lockdown.

### Diet pattern

**Figure 1.**
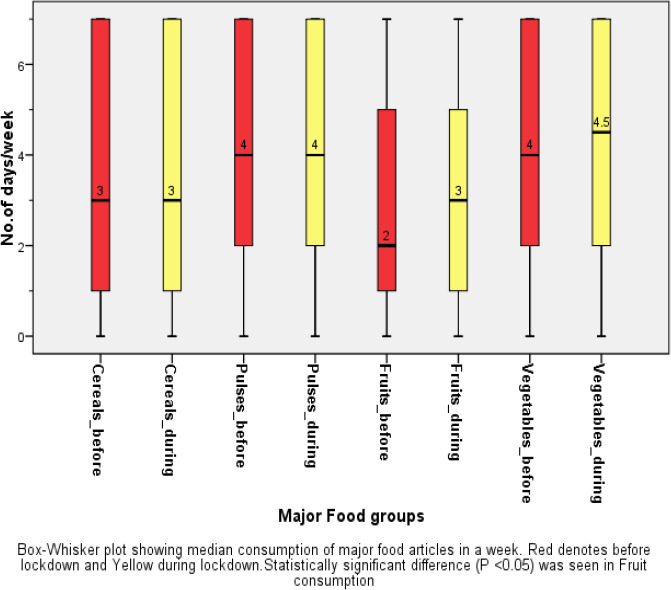
Major food group consumption before & during lockdown (Median days consumption)

**Figure 2.**
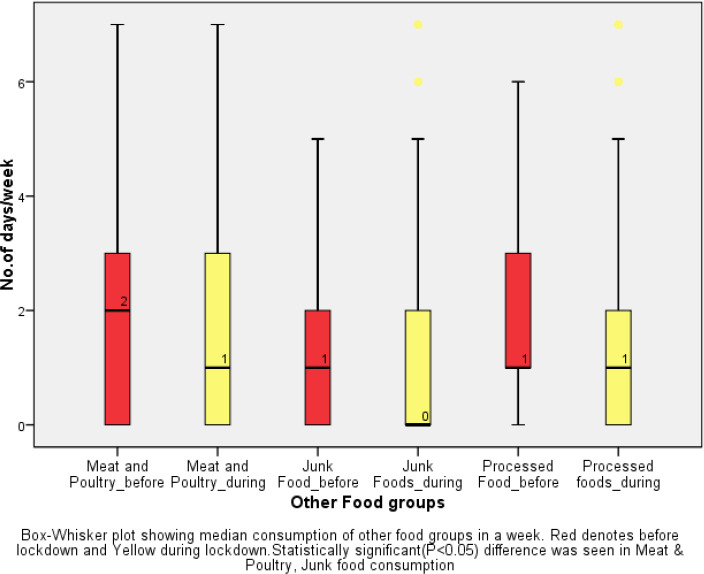
Median days of consumption of other food items (N=622)

The median number of days of consumption of major food groups before and during lockdown among the study group showed significant increase in the consumption of milk and milk products[Median(IQR) days/week-before lockdown: 3(1-7) days; during lockdown: 3(2-7), P<0.001] and Fruits [Median(IQR) days/week -Before lockdown: 2(1-5): During lockdown:3(1-5), P<0.001], and decrease in Meat and Poultry[Median(IQR) days/week-before lockdown: 2(0-3)days: During lockdown: 1(0-3) days, P<0.001],Junk food[Median(IQR) days/week-Before lockdown:1(0-2) days; During lockdown:0(0-2) days, P<0.001]. There was no statistically significant difference in consumption of cereals [Median(IQR) days/week before and during: 3(1-7)days] and pulses[Median(IQR) days/week-before and during lockdown-4(2-7) days].(Figure 1 and 2)

A 7% increase in the often(5-7days) consumption of milk and milk products was reported during lockdown [43.5%] compared to before the lockdown [36.5%], 6.3% increase in fresh fruits consumption [during lockdown:31.5% vs before lockdown: 25.2%], and decrease in often junk food consumption by 4% [during lockdown: 4.9% vs before lockdown: 9%].(Table 4)

A total of 206(33.1%) students reported a midnight craving for food.

### Beverages

**Figure 3.**
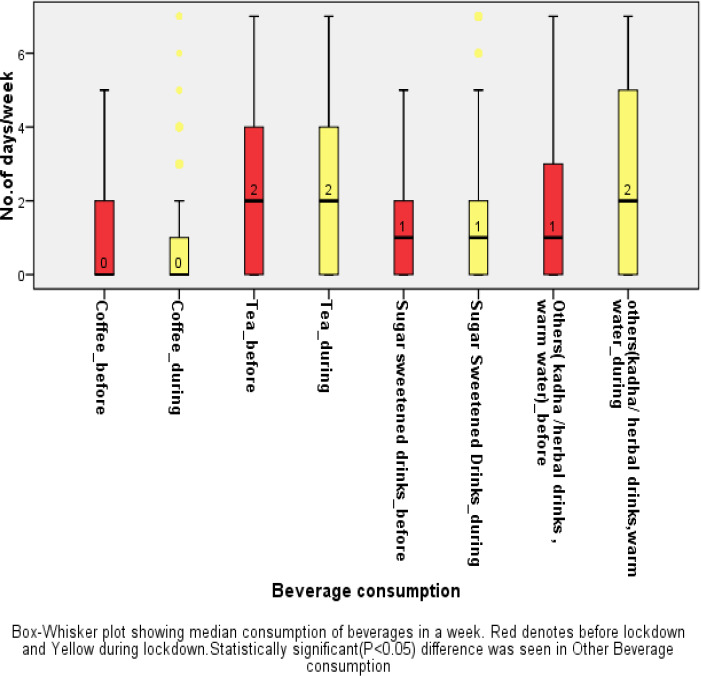
Beverage consumption before and during lockdown(Median days of consumption)

A significant decrease in consumption of sugar sweetened beverages [Median(IQR) days/week- before lockdown: 1(0-3) days; during lockdown: 1(0-2) day, P<0.001] and increase in consumption of other beverages including kadha(herbal drink), warm water[Median(IQR) days/week of consumption :: before lockdown: 1(0-2) day(s) ; during lockdown: 2(0-5) days, P<0.001] was reported by the participants.The consumption of beverages such as coffee[(Median(IQR) days/week of consumption-before lockdown: 0(0-2) day(s) ; during lockdown: 0(0-3) days, P=0.3)] and tea[Median(IQR) days/week of consumption-before lockdown: 2(0-4) day(s) ; during lockdown: 2(0-4) days, P=0.2] were comparable before and during the lockdown.(Figure 3)

There was a statistically significant decrease in often consumption of sugar-sweetened beverages by 1% [before lockdown: 8.5% vs during lockdown: 7.5%, P=0.003] while consumption of other beverages like kadha/herbal drink, warm water has increased by 12.3% during the lockdown.[before lockdown: 14.8% vs during lockdown: 27.2%,P<0.001]. (Table 5)

Perception of students regarding various aspects in their life during lockdown

**Figure 4.**
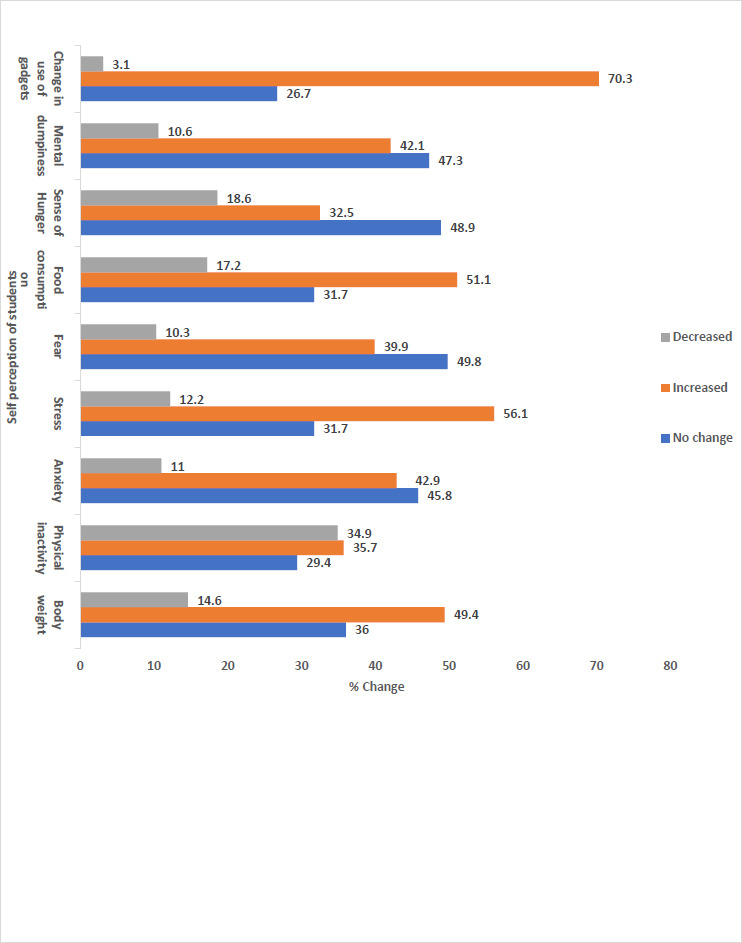
Perception of students regarding various aspects(N=622)

Nearly half, 307(49.4%) of the participants perceived an increase in body weight during the lockdown. Also, more than half, 349 (56.1%) reported having increased stress levels during the lockdown. Nearly half, 318 (51.1%) perceived that their food consumption has increased during the lockdown but only one-third, 202 (32.5%) felt that their sense of hungriness has increased. (Figure 4)

## Discussion

The strict efforts to prevent the spread of the disease, such as social distancing, work from home, school closures, and home isolation/quarantine measures, have a severe impact on human physical and psychological well-being [29].

### Lifestyle changes during lockdown Habits and physical activity

In our study among 622 university students, we saw a significant decrease in the use of tobacco products from 9.2% before lockdown to 6.8% during the lockdown. Similar results were reported in a survey from Italy done during the lockdown [25]. This may be due to the availability of only essential services during the lockdown. Also, our study reported a significant decrease in physical activity from 58.8% before lockdown to 50.2% during the lockdown which was similar to a study by Xiang et al.[30] Nevertheless, our study showed that around half the participants were yet physically active during the lockdown. Another study from the Euromerican population and on Polish adults also showed that more than half (57%) individuals were physically active during the confinement period [31,32]. On the contrary, a study from Italy showed no significant difference in physical activity before and during confinement [25]. This could be attributed to home confinements and the closure of gyms and public parks. Although home workouts with online training are getting popularized, still it took time for people to adapt to this new mode of training for physical activities. As per WHO, moderate-vigorous exercise for at least 150 min per week or a maximum of 5 days per week is recommended [33], our study highlighted that around one third followed physical activity daily and around half followed for almost every day (4-5 days/week) during lockdown which is alarming. This result was better compared to a study from South America who showed only 5.7% of adults were physically active daily and just 25% were for 3-6 days a week [31]. According to research, strong psychological health, a balanced diet, and a healthy lifestyle are critical during pandemics to help the body’s immune system cope with infections like COVID-19 [34,35]. WHO Europe released guidelines and recommendations for healthy eating and staying physically active during lockdowns and home self-quarantine [36].

On calculating the BMI using self-reported weight and height, we found that around 26% of students were in the overweight and obese category (BMI >25 Kg/m2) while a survey from the UK showed that more than half the participants (57%) were in this category and they reported that high BMI was independently associated with lower physical activity [16].

### Sleep pattern

In this study, the participants slept more during lockdown (7.9 hours) as compared to before the lockdown (7.1hrs), and around one-fourth reported waking up late in the morning. A similar finding of about 25-30min increase in night-time sleep and average wake time shift by 30-40 min was reported in a study in the USA [29] and 10% increase in sleeping between 7-9hrs during the lockdown from a university of Iraq [37] which is explained by educational institute shutdown and online mode of classes during lockdown which cut down the traveling time and needs to get up early. Nearly one-fifth (21.8%) of students reported that their sleep pattern was interrupted and one-third (33.9%) noticed no change in sleep which is more compared to a study from Poland which reported no sleep change in around 20% of students [38].

### Gadget use

Nearly more than half (53.2%) of the students reported screen time of >6hours with median(IQR) being 6(4-8) hours in a typical day while Gorincka et al. reported around 40% of students had a screen time of around 4-8 hours.[32] Xiang et al. reported that an increase in screen time of 1730 min/week was seen during the lockdown [30].

### Dietary changes during lockdown Food consumption

In our study, the median (IQR) consumption of cereals and pulses per week before and during lockdown remains the same while there was a significant increase in consumption of fruits, milk, and dairy products during the lockdown and a significant decrease in the consumption of meat and poultry, processed food, and junk food during the lockdown as compared to before lockdown. This could be explained by the fact that rice and wheat are the staple food of East India and so there is no change in consumption before and during lockdown while health-conscious crowd during the COVID-19 pandemic consumed more fruits as there was in news during lockdown that Vitamin C rich fruits help boost immunity and helps fight against the disease [39]. The availability of essential food articles during lockdown explains the decrease in consumption of meat and poultry, junk, and processed food items. A study from Romania and France reported the dietary changes from many countries including France, the USA, China, Italy, Greece showed that there was an increase in consumption of refined sugars and processed food initially during the lockdown but subsequently, there was an increase in fresh food and home-made food consumption including salads and hot beverages [40,41]. Contrarily, a review from Saudi Arabia showed that there is an increased number of meals and unhealthy food such as fast food, sweets and chocolates, carbonated drinks processed meat, and reduced fruits and vegetable consumption during COVID-19 confinement [17].

In our study around one-third of the participants (31.5%) consumed fresh fruits and nearly half (50%) consumed vegetables for at least 5-7 days a week which is marked important norm by the WHO on the consumption of fresh fruits and vegetables to stay healthy and for prevention of NCD [42]. Another cross-sectional study from southern Spain showed that around 60% of participants of age 18-35 years consumed fresh fruits and vegetables daily [43]. Almost 43% of participants consumed cereals, milk and milk products daily which is less compared to a survey in Italy where 64% of participants consumed one portion of cereals daily and around 45% consumed milk and milk products daily [25].

### Beverage consumption

Our study highlighted the increase in consumption of other beverages like Kadha (herbal drinks/hot beverages), warm water in lockdown as compared to before, and little but not significant increase in consumption of sugar-sweetened beverages with no change in coffee and tea consumption before and during the lockdown. Marty et al. also showed the increased consumption of sugar/sweet-tasting beverages [44]. Almost 7% drank coffee and sugar-sweetened beverages (SSB) each and 24% drank tea often(5-7days/week) during the lockdown. While a survey among the youth of China revealed that around 75% drank SSB, around 14% drank coffee and 17% drank tea during home confinement [45].

### Perception about food consumption and weight gain during lockdown

Nearly half the participants (49.5%) felt that they have gained weight during lockdown which was supported by the fact that around 51% felt that their food consumption was increased and about 35% reported an increase in physical inactivity and sense of hunger. A systematic review and meta-analysis from Taiwan showed that lockdown was associated with significant weight gain. (OR 1.93, 95% CI 1.10-1.37) [46]. A study from Southern Spain showed that nearly two-thirds (64%) perceived weight gain and 45% felt reduced physical activities during the lockdown [43] while the findings from Enrique-Martinez et al. were similar to our study in terms of perception of weight gain [31].

It has been reported that stressful situations like pandemics affect the lifestyle and diet of people both positively and negatively [14,47]. Since a balanced diet is an important determinant of the health of an individual [48], these positive and negative changes may have a longer impact than expected on the lives of youths. With the recurrence and subsequent waves of the COVID-19 pandemic, more frequent lockdowns are inevitable and so it is imperative to follow recommended diet and lifestyle policies to prevent a negative health impact.

## Limitation of the study

This is a self-administered online survey and so self-reported bias couldn’t be ruled out. Also, there may be chances of recall bias which couldn’t be eliminated due to study design. Snowball sampling is likely to have resulted in a biased sample. Also, physical activity as metabolic equivalent of task (MET) minutes was not calculated in our study. Other sensitive indicators of nutrition such as Waist hip ratio and the number of servings/day and quantity of particular food group intake were not ascertained in the present study.

## Conclusion

The present study highlighted significant changes in the lifestyle and dietary pattern of university students due to the impact of COVID-19 lockdown. Positive impacts were that there was a significant decrease in the consumption of tobacco products, increase in frequency and proportion of fresh fruits consumption, decrease in the frequency of meat and poultry products, junk food, and sugar-sweetened beverages intake, and increased consumption of herbal drinks and warm water during the lockdown. The observed negative impacts of lockdown were a decrease in physical activity level as per WHO recommendation to prevent NCD and frequency of consumption of milk and milk products, whereas there was a significant increase in sleep duration and use of electronic gadgets.

## Figures and Tables

**Table 1.** Sociodemographic details of the respondents (N=622)

Variable	Categories	N(%)
**Age in years [Mean (SD)]**	23.16(5.1)	
**Gender**	Female	225(36.2)
	Male	397(63.8)
**Stream of education**	Health & allied sciences	297(47.7)
	Science & technology	190(30.5)
	Arts & commerce	95(15.3)
**Family status^#^**	APL	494(79.4)
	BPL	128(20.6)
BMI^*^	Underweight	77(12.4)
	Normal	382(61.4)
	Overweight & obese	163(26.2)

*BMI-Body mass index calculated by self-reported height(cm) and weight (Kg) using formula Weight(kg)/Height(m)2

#APL-Above poverty line BPL-Below poverty line of Indian standrards [28] (self-reported)

**Table 2.** Lifestyle of Students before and during lockdown (N=622)

Variable	Categories	Before Lockdown,N(%)	During lockdown,N(%)	P value
**Tobacco use**	No	565(90.8)	580(93.2)	0.011*
	Yes	57(9.2)	42(6.8)	
**Physical activities^#^**	No	256(41.2)	310(49.8)	<0.001*
	Yes	366(58.8)	312(50.2)	
**Leisure time Sports**	No	281(45.2)	261(41.9)	0.2*
	Yes	341(54.8)	361(58.1)	
**Sleep duration(in Hours) [Mean(SD)]**		7.1(1.1)	7.9(1.7)	P=<0.001**

**Table 3.** Sleep characteristics during Lockdown(N=622)

Sleep attributes		N	%
**Sleep cycle**	Sleeping late night	310	49.8
	Waking up late	167	26.8
	Excessive daytime sleeping	96	15.4
**Sleep pattern**	Continuous	444	71.4
	Interrupted	136	21.8
	Difficult to fall asleep due to fear of COVID-19	42	6.8
	No change in the pattern	211	33.9

**Table 4.** Distribution of study participants according to major food consumption before and during lockdown (N=622)

Major Food group	Frequency (in a week)	N(%)	N(%)	
		Before Lockdown(1)	During Lockdown(2)	Difference (2-1)
**Cereals**	**Never**	64(10.3)	78(12.5)	14(2.2)
	**Some days (1-4 days)**	294(47.3)	275(44.2)	-19(3.1)
	**Often (5-7days)**	264(42.4)	269(43.2)	5(0.08)
**Pulses**	**Never**	20(3.2)	29(4.7)	9(1.5)
	**Some days(1-4days)**	311(50)	301(48.4)	-10(1.6)
	**Often(5-7days)**	291(46.8)	292(46.9)	-1(0.01)
Milk and Milk products*	**Never**	60(9.6)	36(5.9)	-24(3.8)
	**Some days (1-4days)**	335(53.9)	315(50.6)	-20(3.2)
	**Often (5-7days)**	227(36.5)	271(43.5)	44(7)
Meat & Poultry*	**Never**	178(28.6)	219(35.3)	41(6.6)
	**Some days (1-4days)**	401(64.5)	359(57.7)	-42(6.7)
	**Often (5-7days)**	43(6.9)	44(7)	1(0.01)
Fruits*	**Never**	69(11)	58(9.3)	-11(1.8)
	**Some days(1-4days)**	396(63.6)	368(59.2)	-28(4.5)
	**Often(5-7days)**	157(25.2)	196(31.5)	39(6.3)
**Vegetables**	**Never**	16(2.6)	18(2.9)	2(0.03)
	**Some-days (1-4days)**	298(47.9)	293(47.1)	-5(0.08)
	**Often (5-7days)**	308(49.5)	311(50)	3(0.04)
Processed Foods*	**Never**	155(24.9)	215(34.6)	60(9.6)
	**Some days (1-4days)**	373(59.9)	336(54)	-37(5.9)
	**Often (5-7days)**	94(15.2)	71(11.4)	-23(3.7)
Junk Food*	**Never**	203(32.6)	322(51.8)	-119(19.1)
	**Some days (1-4days)**	363(58.4)	269(43.3)	-94(15.1)
	**Often (5-7days)**	56(9)	31(4.9)	-25(4)

*Statistically significant by McNemar-Bowker test

**Table 5.** Distribution of study participants according to Beverage consumption before & during Lockdown (N=622)

Beverage	Frequency(in a week)	N(%)	N(%)	
		Before Lockdown(1)	During Lockdown(2)	Difference (2-1)
**Coffee**	**Never**	335(53.8)	361(58)	26(4.1)
	**Some days(1-4days)**	244(39.3)	216(34.7)	-28(4.5)
	**Often(5-7days)**	43(6.9)	45(7.3)	2(0.3)
Tea*	**Never**	196(31.5)	179(28.7)	-17(2.7)
	**Some days(1-4days)**	286(45.9)	293(47.1)	7(1.1)
	**Often(5-7days)**	140(22.6)	150(24.2)	10(1.6)
Sugar-sweetened beverages*	**Never**	229(36.8)	294(47.3)	65(10.5)
	**Some days(1-4days)**	340(54.7)	281(45.2)	-59(9.4)
	**Often(5-7days)**	53(8.5)	47(7.5)	-6(0.9)
Others (Kadha/Herbal drink/Warm water)*	**Never**	289(46.5)	157(25.2)	-132(2.1)
	**Some days(1-4days)**	241(38.7)	296(47.6)	55(8.8)
	**Often(5-7days)**	92(14.8)	169(27.2)	77(12.3)

*Statistically significant by McNemar-Bowker test
